# Explainable machine learning for stroke risk prediction: a comparative study with SHAP-based interpretation

**DOI:** 10.3389/fneur.2025.1716984

**Published:** 2026-01-12

**Authors:** Xiaoyu Tang, Min Tang, Wu Liu, Shaoyang Cui

**Affiliations:** 1 Shenzhen Hospital (Fu Tian) of Guangzhou University of Chinese Medicine, The Sixth Clinical School, Guangzhou University of Chinese Medicine, Shenzhen, Guangdong, China; 2 The Sixth Clinical Medical School, Guangzhou University of Chinese Medicine, Guangzhou, Guangdong, China; 3 South China Research Center for Acupuncture and Moxibustion, Medical College of Acu-Moxi and Rehabilitation, Guangzhou University of Chinese Medicine, Guangzhou, Guangdong, China; 4 College of Acupuncture and Orthopedics, Hubei University of Chinese Medicine, Wuhan, Hubei, China

**Keywords:** machine learning, model interpretability, neural network, SHAP, stroke prediction

## Abstract

**Background:**

Stroke is one of the leading causes of death and disability worldwide, making early screening and risk prediction crucial. Traditional methods have limitations in handling nonlinear relationships between variables, class imbalance, and model interpretability.

**Methods:**

Logistic regression (LR), random forest (RF), extreme gradient boosting (XGBoost), categorical boosting (CatBoost), multi-layer perceptron (MLP) neural network, and ensemble models were constructed and compared. Their performance in stroke risk prediction was systematically evaluated, and feature contributions were interpreted using SHapley Additive exPlanations (SHAP). Confusion matrices and Precision-Recall (PR) curves were used to compare the differences in recognition of the positive class (stroke patients) among the models, and training time was calculated to quantify resource consumption.

**Results:**

The ensemble model and neural network demonstrated superior overall predictive ability to traditional algorithms, with the MLP performing particularly well in terms of recall. SHAP results revealed that “hypertension,” “average blood glucose level,” and “age” were key influencing factors. Confusion matrices and PR curves indicated differences in positive classification among the models. Training time analysis provided a basis for resource assessment for subsequent deployment.

**Conclusion:**

Machine learning methods have advantages in stroke risk prediction. Incorporating interpretability analysis can enhance the clinical credibility of the models, providing data and methodological reference for stroke risk stratification management and early warning.

## 1 Introduction

Stroke is an acute cerebrovascular event characterized by ischemia or hemorrhage of brain tissue and is one of the leading causes of death and disability worldwide (1). According to statistics from the World Health Organization, stroke causes more than 6 million deaths each year and causes tens of millions of patients to lose their ability to take care of themselves for a long time, which seriously threatens human health and social development (2, 3). As a country with a high incidence of stroke, my country has a continuously increasing incidence rate due to factors such as population aging and metabolic syndrome. In particular, ischemic stroke accounts for more than 80% of the total number of strokes, and early identification and early warning intervention are particularly important. However, due to the high heterogeneity of clinical manifestations of stroke and the frequent presence of multiple risk factors (such as hypertension, diabetes, smoking, age, socioeconomic status, etc.), the traditional stratified prediction strategy that relies on a single risk factor has problems such as insufficient sensitivity and poor individual adaptability. There is an urgent need for more accurate and comprehensive data-driven prediction methods to assist stroke risk management (4, 5).

In recent years, the application of machine learning (ML) technology in medical predictive modeling has developed rapidly. Its core advantage is that it can automatically learn complex nonlinear mapping relationships between features from historical data without explicitly modeling clinical pathways and causal mechanisms (6–8). Compared with traditional linear regression methods, ML models such as random forests, gradient boosting trees (such as XGBoost, LightGBM), and artificial neural networks (ANN) have stronger generalization and feature expression capabilities (9–11). Random forests effectively reduce the risk of overfitting by integrating multiple decision trees and introducing feature and sample randomness (12, 13). Gradient optimization-based integrated models (such as XGBoost) gradually correct residuals to improve the robustness of the model while maintaining accuracy (14–16). In addition, neural network structures such as multi-layer perceptrons (MLPs) can learn high-order nonlinear feature interactions and show significant advantages in processing large-scale, multi-dimensional, and heterogeneous health data (17–19).

Although existing studies have shown that machine learning methods have significant advantages in stroke risk prediction, current research still faces two core scientific challenges: first, the coexistence of data category imbalance and high-order nonlinear relationships between variables results in limited model identification of high-risk stroke individuals; second, high-performance models have poor interpretability, which limits their credibility and applicability in clinical practice (20–24). Specifically, stroke samples account for a very low proportion of most real data, and traditional models tend to overfit the majority class and ignore the accurate identification of minority stroke classes. At the same time, stroke is affected by multiple factors (such as age, blood sugar, BMI, heart disease, etc.), and the complex nonlinear and interactive effects between variables make it difficult to express complete risk information through linear models or single features (25–27). In addition, although complex models such as XGBoost and neural networks can effectively improve prediction performance, they are widely regarded as “black boxes” and lack clinical explanations for individual predictions, which seriously restricts their credibility and adoptability in medical practice (28–32).

**Figure 1:**
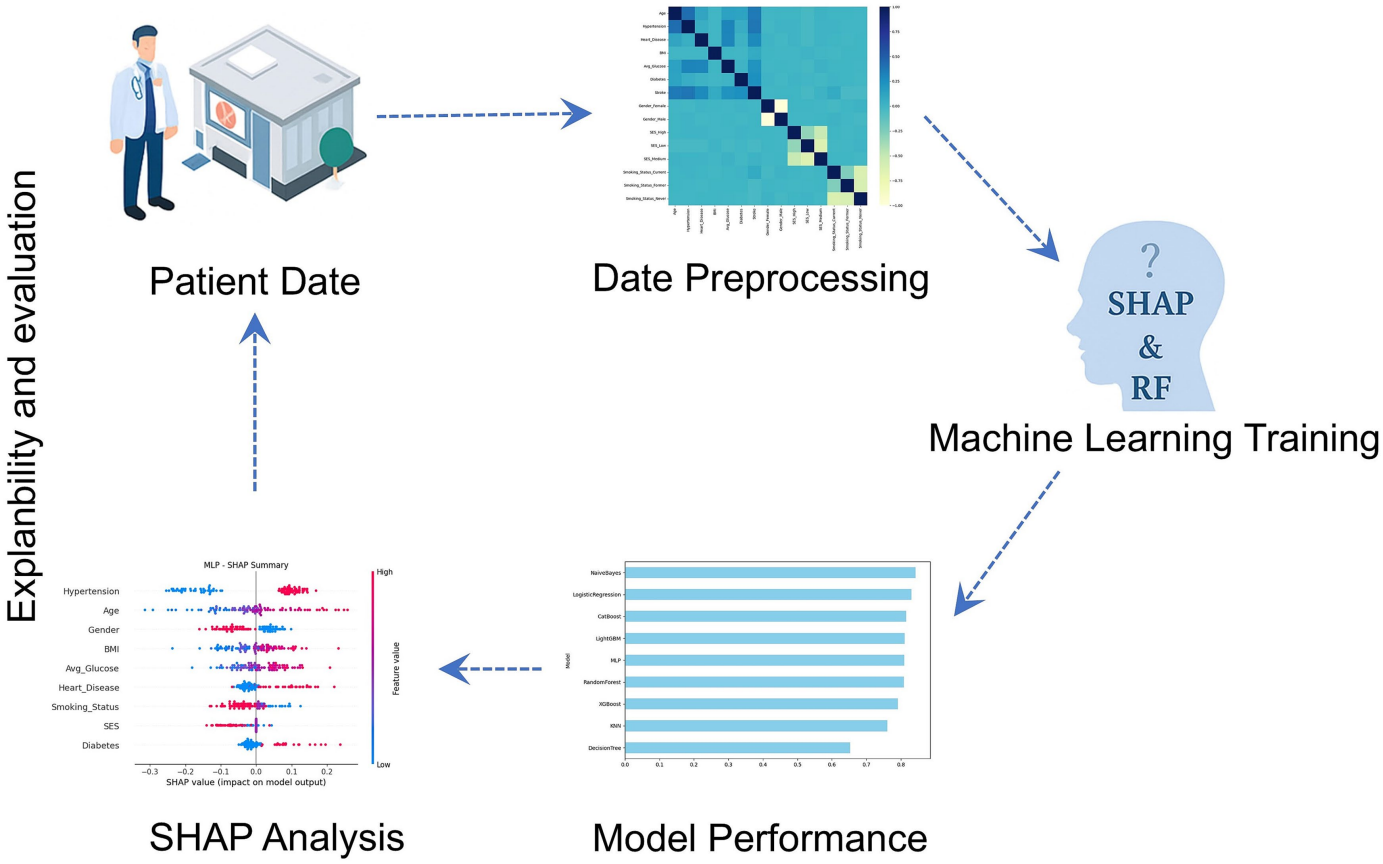
Research flow chart. This figure shows the machine learning modeling process of this study, including key steps such as data preprocessing, SMOTE oversampling, model training, evaluation and interpretation.

In response to the above problems, this study constructed a multi-dimensional evaluation framework integrating “modeling performance-interpretability-resource complexity” (Figure 1). Based on the public stroke diagnosis dataset, the prediction performance and explanation ability of various mainstream models were systematically compared. The SMOTE algorithm was introduced to oversample the minority samples of stroke to alleviate the impact of class imbalance on model stability. Various classic and advanced models such as logistic regression, random forest, XGBoost, CatBoost, MLP, etc. were constructed and compared to evaluate their comprehensive performance under key indicators such as Accuracy, Recall, and area under curve (AUC). The SHAP interpretation framework was used to quantify the marginal contribution of key features, and the SHAP summary graph, Top-N feature bar graph and individual force plot were further drawn to achieve clinical interpretation of model output. The bias and risk of the model in stroke recognition were explored through confusion matrix, PR curve and other methods to enhance its medical adaptability. This study not only systematically compared the performance differences of mainstream machine learning (ML) models in stroke prediction tasks, but also improved the scientificity, transparency and clinical usability of the models by introducing explanatory analysis and class imbalance processing mechanisms, providing a theoretical basis and practical support for building a reliable personalized stroke prediction system.

## 2 Materials and methods

### 2.1 Data

The data used in this study comes from the publicly available “Stroke Diagnosis and Health Metrics Data”^1^ on the Kaggle platform. This dataset contains 10,000 individual records, covering health indicators and lifestyle information such as age, sex, hypertension, heart disease, average glucose level (avg glucose level), BMI, smoking status, marital status, and socioeconomic status (SES). The definition, data type, and category distribution of each variable in the dataset are shown in the Supplementary Table 1. Inclusion criteria were age greater than 18 years, complete records of the outcome variable (Stroke), and complete records of key health indicators (such as age, average glucose level, and BMI). Of the 10,000 samples, 2,978 were stroke-positive (Stroke = 1), accounting for 29.78%, while 7,022 were non-stroke (Stroke = 0), indicating a certain degree of class imbalance. To mitigate the impact of class imbalance on model training, this study introduced the SMOTE method for oversampling during the training phase.

### 2.2 Data preprocessing

In order to improve the modeling effect and meet the input requirements of the machine learning algorithm, the following preprocessing steps were performed on the original data.

*Missing value processing*: missing BMI values were filled with the median.

*Category variable encoding*: One-Hot Encoding was used to process categorical variables including gender, job type, smoking status, etc.

*Feature standardization*: Z-score standardization was performed on continuous variables (such as age, avg. glucose level, BMI).

*Class imbalance handling*: This study uses SMOTE to upsample the training data. The specific upsampling operation is automatically completed through the pipeline in the cross-validation process, and only applies to the training subset in each fold, not to the validation or test set, to avoid information leakage.

### 2.3 Model and parameter adjustment

This study constructed a variety of models including traditional machine learning, integrated methods, deep learning and AutoML, covering basic models [logistic regression (LR), K nearest neighbor (KNN), decision tree (DT), naive Bayes (NB)], integrated models [random forest (RF), XGBoost, LightGBM, CatBoost], deep neural networks [multi-layer perceptron (MLP) integrated strategies soft voting, hard voting (Voting), Stacking].

In terms of model parameter adjustment, GridSearchCV grid search or Bayesian optimization method is used to adjust hyperparameters for integrated models to improve model performance. All models use the same data partitioning strategy and evaluation indicators to ensure consistency and fairness of comparison.

Furthermore, to ensure the transparency and reproducibility of the model training process, this study established explicit hyperparameter search ranges for all models and selected the optimal parameter combinations using 5-fold hierarchical cross-validation. The specific search ranges and final selected parameters for different model types (traditional machine learning, ensemble models, deep learning models, and ensemble strategies) are detailed in Supplementary Table 2. All models underwent preprocessing, SMOTE, and training steps through a unified pipeline to ensure consistency and fairness in the training process.

This study AutoML experiments are implemented based on the AutoGluon-Tabular (v0.x) framework. AutoML automatically trains and evaluates multiple candidate algorithms, including linear models, tree models (RF, XGBoost, LightGBM, CatBoost), and neural network models, by automatically searching for different model structures and hyperparameter combinations. This study sets a maximum runtime of 30 min for AutoML, allowing the framework to automatically select the optimal model structure and hyperparameters within this time budget. AutoML’s evaluation is based on 5-fold hierarchical cross-validation consistent with other models, using only the training set for search and hyperparameter tuning, and finally performing a one-time validation on an independent test set to ensure fair comparison. The final output of AutoML is the best-performing model selected within the framework (usually a gradient boosting tree model), combined with an automatic multimodel ensemble strategy to further improve performance.

### 2.4 Model evaluation strategy

To systematically evaluate the predictive performance and generalization ability of each model, this study divides the complete dataset into training and test sets in an 8:2 ratio. The test set remains completely independent throughout the training process and is used only for final performance evaluation.

During the training phase, all models undergo 5-fold stratified cross-validation (CV) only on the training set. To avoid information leakage and ensure process consistency, feature preprocessing (including numerical variable standardization and one-hot encoding of categorical variables) and SMOTE upsampling are managed through a pipeline. SMOTE is performed only within the training subset of each fold and does not affect the validation fold or the final test set, thus ensuring model independence and evaluation fairness.

In cross-validation, metrics such as AUC, PR-AUC, F1, and Recall are recorded, and the model’s robustness is reported in the form of mean ± standard deviation (mean ± SD). After cross-validation is completed during the training phase, the final model undergoes a one-time independent validation on the test set (which was not used in the training). Visualization methods included bar charts, ROC curves, and PR curves; further, the classification details of the models were analyzed using a confusion matrix.

In addition, this study calculated the Brier Score as an overall calibration metric on an independent test set; a lower Brier Score indicates higher consistency between the model’s predicted probability and the actual stroke incidence.

To ensure fairness in model comparisons, this study employed the same training-validation-testing process for all models (including LR, RF, XGBoost, LightGBM, CatBoost, MLP, Stacking, AutoML, and TabNet). All models were built on the same training set, internally evaluated using consistent 5-fold hierarchical cross-validation, and finally tested on completely independent test sets. No test sets were used for parameter tuning or model selection throughout the model development process, thus avoiding data leakage and ensuring the reproducibility and fairness of performance comparisons.

### 2.5 Interpretability analysis method

To improve the transparency and clinical interpretability of the model, this study employed the SHAP (SHapley Additive exPlanations) method to analyze the contribution of each feature in the model’s predictions. The global feature importance and ranking were presented through SHAP summary plots and bar charts of the average |SHAP| values of the features. Individual-level interpretations were then performed on typical samples based on force plots to reveal how different features influence individual prediction results. This interpretive analysis helps identify the most critical variables in the model’s decision-making and examines their consistency with clinical understanding.

At the implementation level, this study selected different types of SHAP interpreters based on the structural characteristics of different models: for tree models (such as XGBoost, LightGBM, CatBoost, and Random Forest), TreeExplainer was used to obtain Shapley values consistent with the tree structure and with high computational efficiency; for deep neural network models (MLP), due to their non-tree structure, KernelExplainer was used, and 100 samples were randomly selected from the training set as background data to construct the SHAP baseline distribution, achieving a balance between interpretive accuracy and computational efficiency. Furthermore, this study calculated the SHAP interaction value to uncover potential nonlinear feature interactions, and the calculation was also based on the aforementioned interpreter and background sample settings.

## 3 Results

### 3.1 Correlation structure

**Figure 2:**
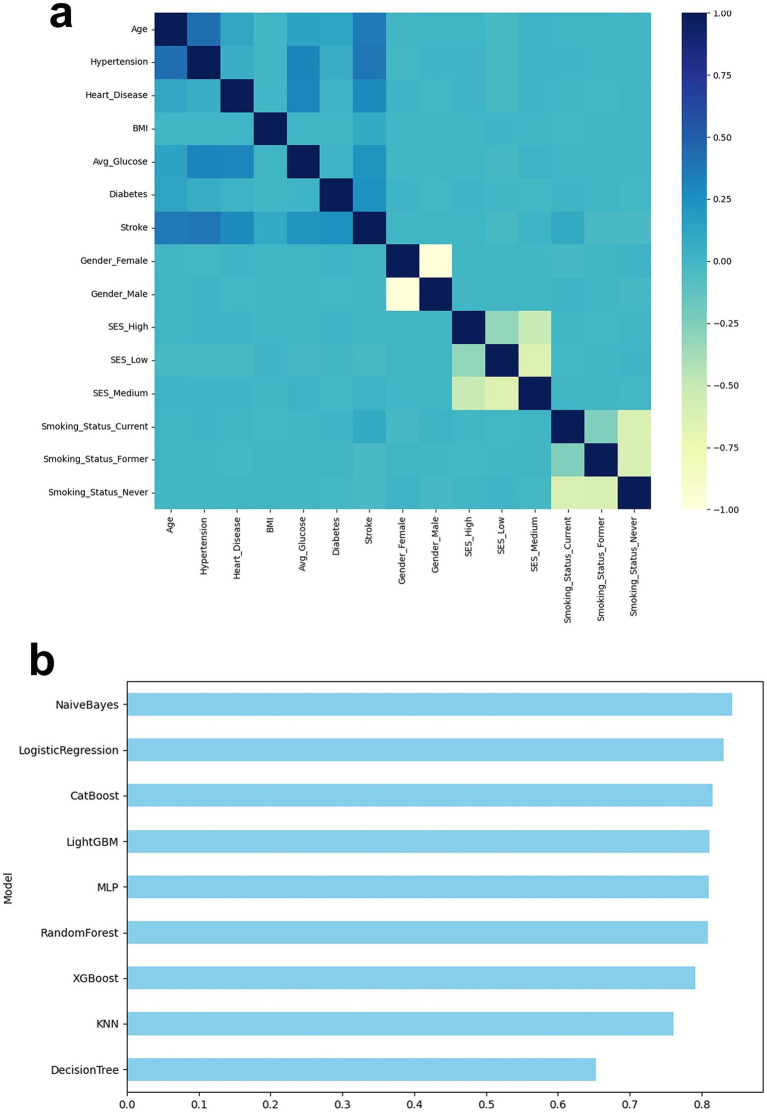
**(a)** Variable correlation heat map; **(b)** Model performance bar graph (Accuracy, Recall, F1, AUC).

To intuitively understand the relationship between features, a Pearson correlation heat map of all variables in the stroke dataset was drawn (Figure 2a; Supplementary Table 3). Overall, age was moderately positively correlated with Hypertension and heart disease (r ≈ 0.5), which is consistent with clinical observations: with age, the risk of hypertension and cardiovascular disease increases significantly, which may be related to atherosclerosis, endothelial dysfunction and long-term metabolic load (33). Age was also positively correlated with diabetes and avg. glucose, suggesting that aging is accompanied by glucose metabolism disorders, which is an important dimension for assessing stroke risk.

Among the variables related to lifestyle, smoking status was significantly negatively correlated with the SES encoding dimension, mainly due to the mutually exclusive property of one-hot encoding, but it also reflects the law of social behavior to a certain extent: low SES people have a higher smoking rate and an increased risk of chronic diseases. This phenomenon has been widely evidenced in epidemiological studies (34). Furthermore, a mild positive correlation was observed between BMI and avg. glucose, suggesting that obesity and impaired glucose metabolism may contribute to stroke risk, consistent with the pathogenic hypothesis underlying the metabolic syndrome pathway. Most intervariate correlation coefficients ranged from −0.4 to +0.5, with no significant multicollinearity (|r| > 0.9). Information redundancy was minimal, suggesting favorable modeling conditions. The overall correlation between stroke and various variables was low, suggesting that its prediction may rely on complex interactions among multiple features rather than a single strong factor. Therefore, the use of nonlinear machine learning modeling combined with explanatory analyses such as SHAP is necessary.

Figure 2b summarizes the performance of each model across four metrics. Accuracy, Recall, F1, and AUC. The overall trend is consistent with the subsequent ROC/PR analysis: ensemble and regularized models (such as LightGBM, XGBoost, and Logistic Regression) excel in overall discriminability and robustness; MLP, in particular, exhibits outstanding recall, making it more effective at detecting positive individuals. This is corroborated by the confusion matrix results: LightGBM achieves the highest F1 of 0.74, while XGBoost and Logistic Regression achieve F1s of 0.72 and 0.71, respectively. Decision Tree and KNN, however, achieve F1s of 0.61 and 0.64, respectively, indicating relatively weak overall performance. This bar chart visually illustrates the trade-offs among different models in terms of overall accuracy, positive identification, and overall balance (F1), providing a reference for subsequent scenario-specific model selection.

### 3.2 Model discriminative performance

**Figure 3:**
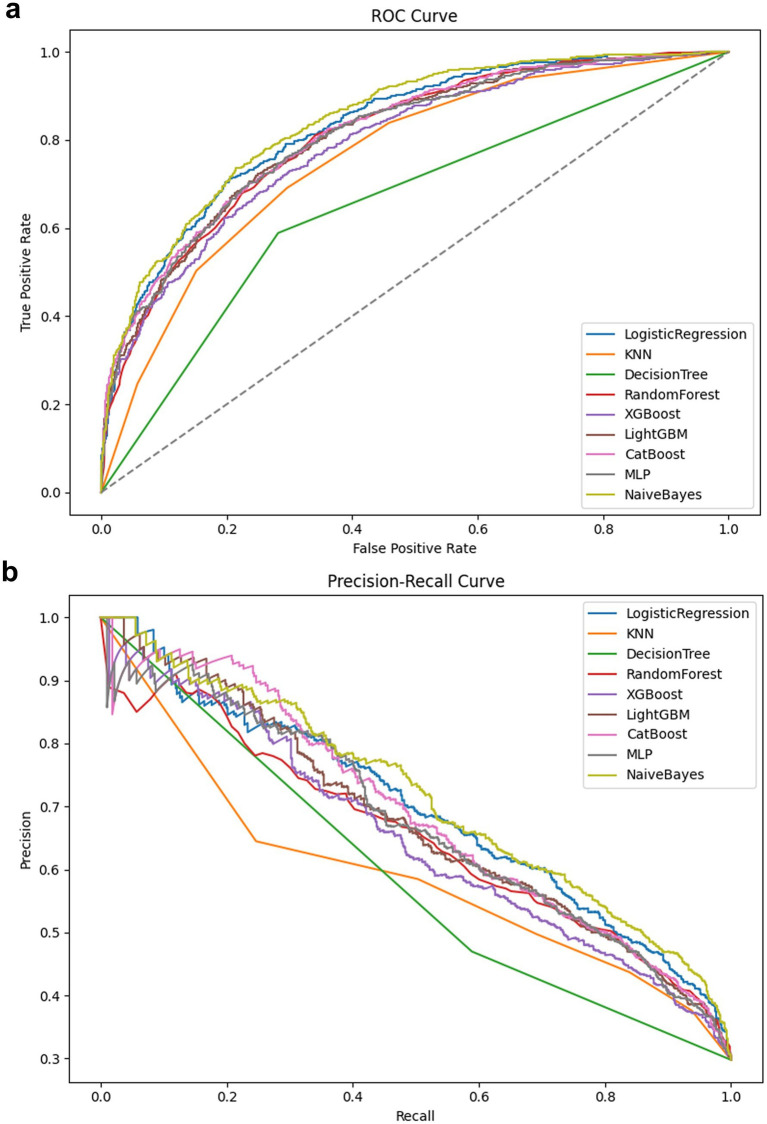
**(a)** Comparison of ROC curves of multiple models; **(b)** PR curve.

Multi-model ROC (Figure 3a) and PR (Figure 3b) are plotted to assess overall discriminative ability and positive identification performance in the context of class imbalance, respectively. The AUC of the ROC reflects the overall distinction between positive and negative classes; the PR focuses more on the accurate identification of positive (stroke) samples.

Figure 3a and Supplementary Table 4 shows that Logistic Regression (AUC = 0.84), Random Forest (AUC = 0.82), XGBoost (AUC = 0.82), and LightGBM (AUC = 0.83) all have high discriminative power, with their curves close to the upper left corner, indicating good generalization ability for risk identification while balancing sensitivity and specificity. XGBoost and LightGBM, as gradient boosting ensemble algorithms, are better at capturing nonlinearities and high-order interactions, and perform stably in high-dimensional feature spaces. It’s important to note that while Naive Bayes achieved a high ROC (AUC) of 0.84, it performed relatively poorly in PR (AP = 0.70), suggesting the existence of “ROC illusion”: in severely class-imbalanced datasets, ROC is insensitive to misclassification of the minority class and tends to overestimate model performance. In addition, to further evaluate the robustness of the models and reduce the risk of overfitting, we performed 5-fold stratified cross-validation on all models on the training set. The 5-fold mean ± standard deviation of AUC, PR-AUC, F1, and Recall can more comprehensively reflect the stability of the models under different training data partitions (Supplementary Table 5).

Figure 3b further shows that the average precision (AP) in the PR space is generally lower than the corresponding AUC, indicating that most models experience some accuracy loss in imbalanced scenarios. Among them, Logistic Regression (AP = 0.71) and LightGBM (AP = 0.70) exhibit strong positive recognition capabilities, maintaining high accuracy particularly in the medium-to-high recall range. MLP (AP = 0.66) and XGBoost (AP = 0.67) performed second best, with fluctuations in accuracy in the low-recall range, potentially due to insufficient calibration of the probability output or instability on extreme samples. Decision Tree (DT, AP = 0.41) and KNN (AP = 0.57) performed relatively poorly. The former is prone to overfitting, while the latter is insensitive to distribution in high-dimensional spaces and suffers from poor discrimination.

To supplement the model’s discriminative ability, this study further calculated the Brier Score of the final model (XGBoost Pipeline) based on an independent test set to evaluate the consistency between the predicted probability and the actual stroke incidence. The results showed that the model’s Brier Score was 0.1399, indicating a moderate degree of consistency between its risk probability output and the actual outcome. Although the model’s discriminative performance is relatively excellent, the Brier Score suggests that its probability output may still exhibit some degree of overconfidence or underconfidence, which needs to be considered when using the model for clinical risk assessment.

In summary, ROC and PR provide complementary assessments: the former emphasizes overall discrimination, while the latter emphasizes the reliability of minority class predictions. The results suggest that ensemble and regularized models, such as LightGBM, XGBoost, and Logistic Regression, have strong discriminative power and clinical applicability in imbalanced medical scenarios. Future work could further enhance the usability and reliability of these models by combining probability calibration, class resampling, and interpretability enhancements (e.g., SHAP/LIME).

### 3.3 Confusion matrix

**Figure 4:**
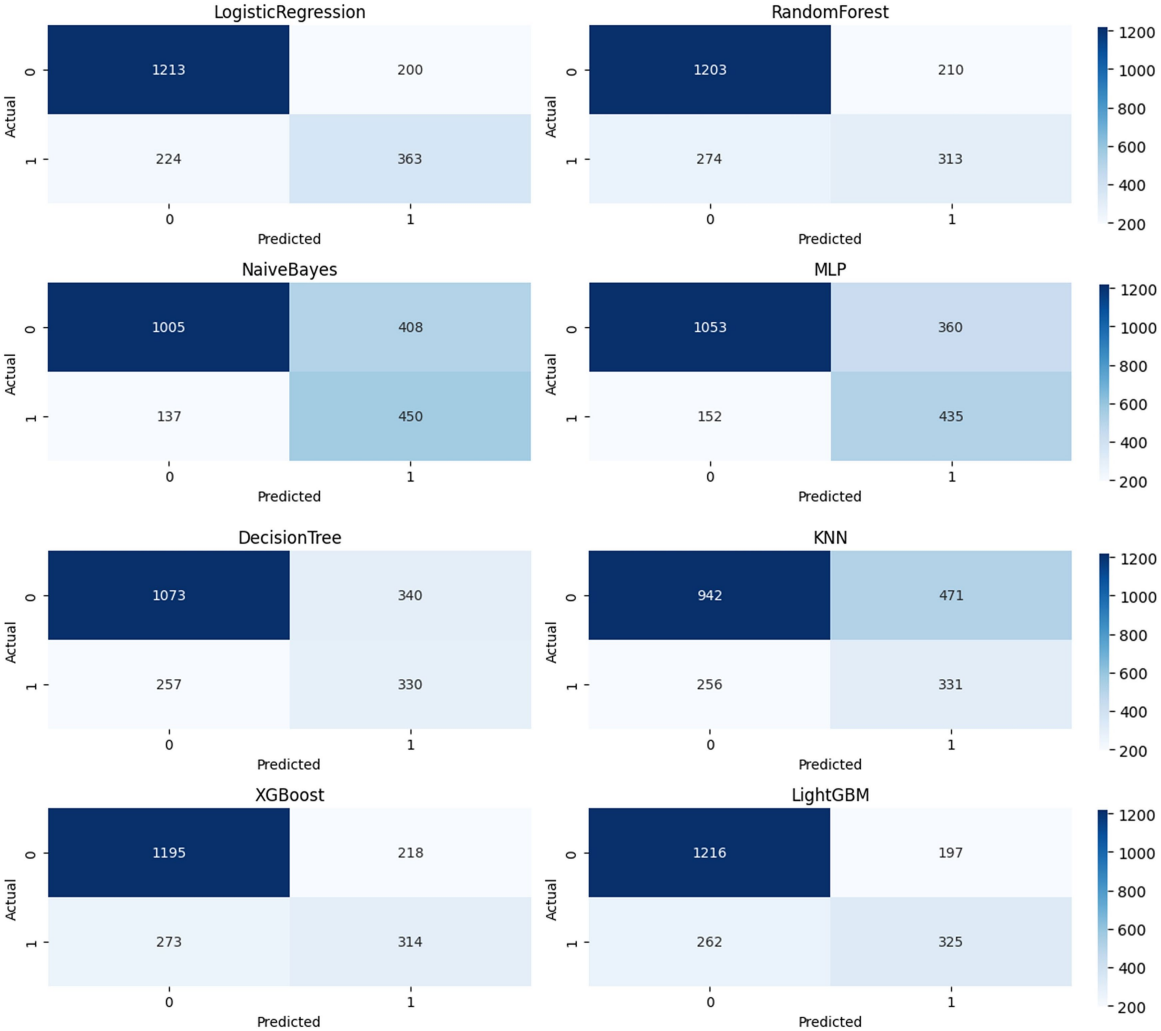
Confusion moment comparison chart.

The confusion matrices (Figure 4) of eight models (LR, RF, DT, KNN, NB, MLP, XGBoost, and LightGBM) were compared at a fixed threshold (typically 0.5) to evaluate their classification performance in a realistic clinical scenario.

LightGBM achieved a good balance between identifying stroke samples and limiting false positives (TP = 325, FP = 197), with a high number of true negatives (TN = 1,216), demonstrating high overall accuracy and stability. Logistic Regression and XGBoost also achieved satisfactory TP (363 and 314, respectively), but the former had a higher FP = 200 and the latter had a slightly higher FN = 273, suggesting room for improvement in identifying borderline samples. KNN and Naive Bayes showed significant bias in the confusion matrix: KNN’s FP = 471 was high, potentially leading to false positives for non-stroke patients; Naive Bayes’ FN = 137 was lower, but at the expense of FP = 408, which could lead to unnecessary intervention and resource waste in real-world scenarios. Decision Tree (DT) showed the weakest overall balance (TP = 330, FP = 340), with limited expressive power in high-dimensional nonlinear spaces. MLP achieved a compromise between precision and recall (TP = 435, FP = 360), demonstrating the potential of neural networks for modeling complex variable relationships. Further calculations showed that LightGBM outperformed the other models with an F1-score of 0.74; XGBoost and Logistic Regression achieved scores of 0.72 and 0.71, respectively; and Decision Tree and KNN achieved scores of 0.61 and 0.64, respectively, suggesting insufficient ability to distinguish stroke from non-stroke samples. The confusion matrix provides a clinically relevant diagnostic perspective. Combined with ROC and PR, it forms a three-dimensional evaluation framework, providing a basis for establishing a highly reliable and feasible data-driven stroke prediction model.

### 3.4 Feature importance and interpretability

**Figure 5:**
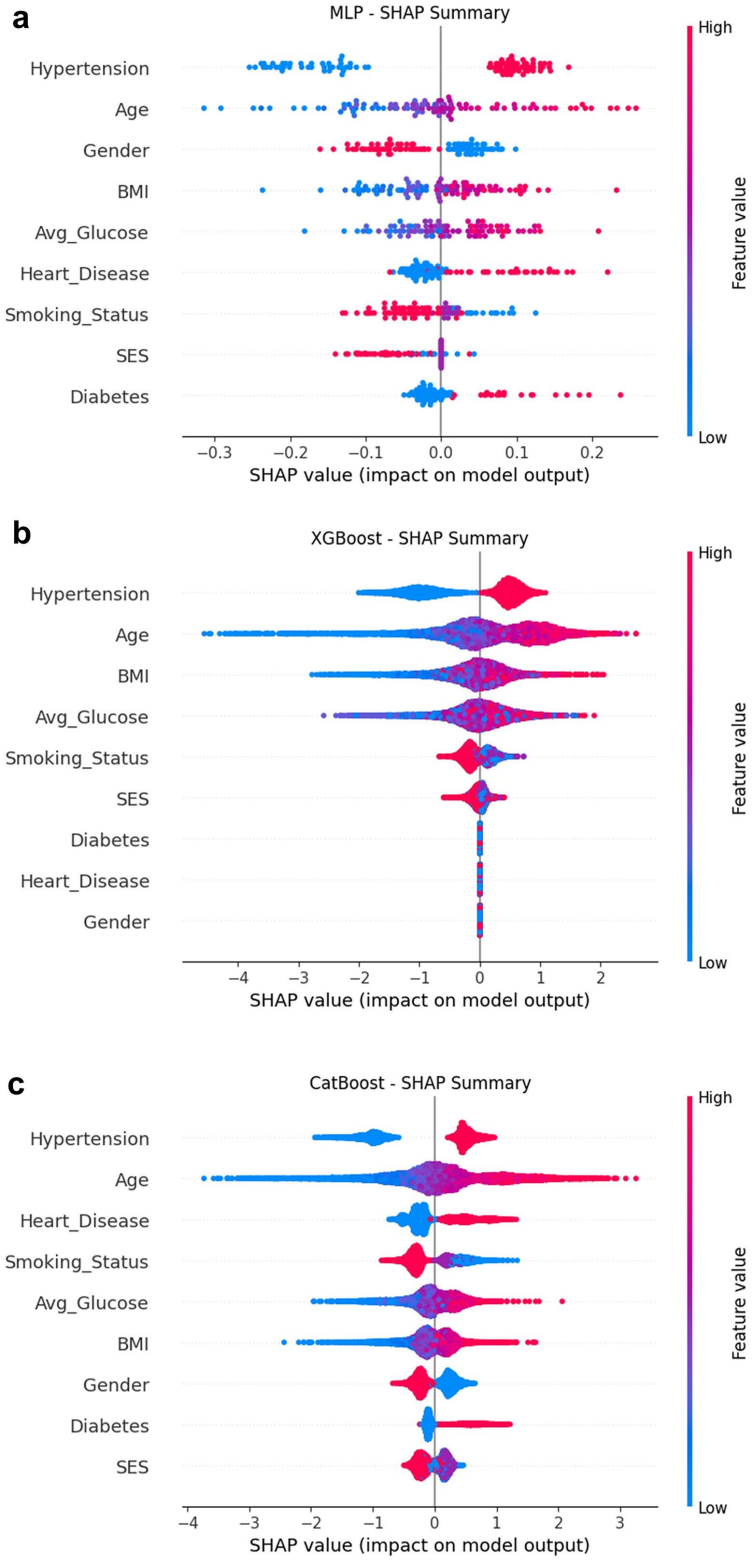
SHAP summary composite image. **(a)** MLP; **(b)** XGBoost; **(c)** CatBoost.

**Figure 6:**
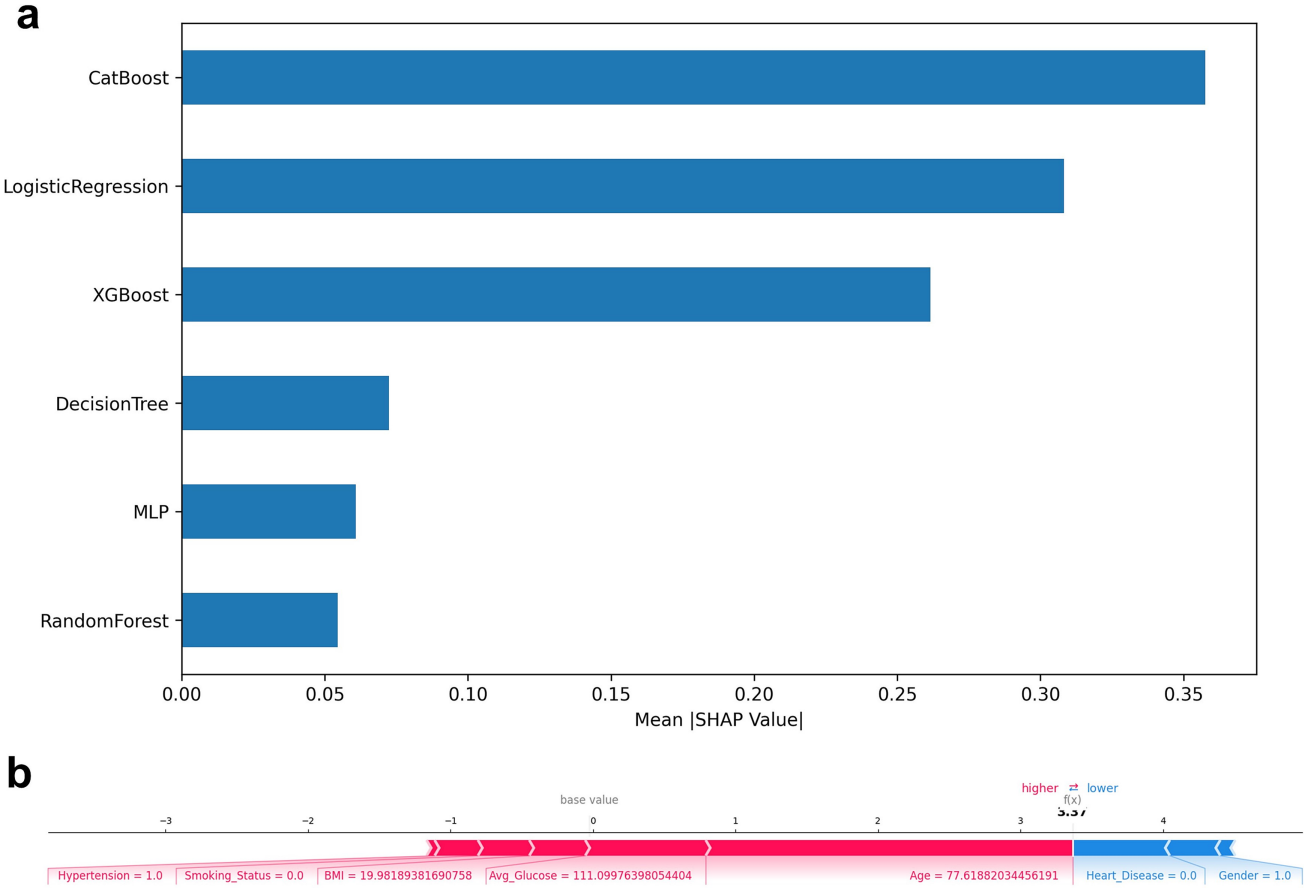
**(a)** Top-N average SHAP value histogram; **(b)** SHAP force plot.

SHAP was used to interpret four representative models (Figures 5, 6a): CatBoost, RF, XGBoost, and MLP. The top five features of CatBoost are age, hypertension, avg. glucose level, heart disease, and BMI, which are highly consistent with previous clinical studies (35). MLP assigns higher weights to social behavioral features such as smoking status and SES, suggesting the potential advantages of deep models in nonlinear learning; XGBoost distinguishes work type and residence type more strongly, indicating the role of living environment and occupation type. This ranking difference reflects the difference in the ability of different models to model variable interactions, providing a reference for subsequent medical mechanism research and model integration optimization. SHAP helps identify typical risk factors and suggests potential physiological pathways (such as the effect of SBP on cerebral vascular endothelial stress), which helps to enhance clinical trust.

A SHAP plot of a typical individual (Figure 6b) was constructed to demonstrate the causal contribution of a single “stroke/non-stroke” prediction: individuals predicted as “stroke” were driven by risk factors such as advanced age (>70 years), diabetes, history of hypertension, and higher BMI; individuals predicted as “non-stroke” were dominated by protective factors such as young age, absence of underlying diseases, and a healthier metabolic state (hypoglycemia). This type of output provides clinicians with intuitive evidence of “why the model makes such a judgment,” improving transparency and adoptability (36).

Meanwhile, we explored the synergistic relationships between features using SHAP interaction values and identified the top 10 most strongly interacting variable combinations (Supplementary Table 6) (37). Among the top 10 most significant feature pairs, hypertension–heart disease had the highest interaction strength (0.086), suggesting that when hypertension and heart disease coexist, the risk of stroke may have an additive or even amplifying effect. The interaction between age–heart disease and Age–Hypertension was also among the highest, indicating that age further increases the probability of stroke on top of these cardiovascular risk factors, consistent with clinical observations that stroke is more common in elderly patients with hypertension and heart disease. Among metabolic factors, BMI–avg glucose (0.030) and age–avg glucose (0.025) showed a moderate-strength interaction, suggesting that elevated blood glucose and weight gain may jointly influence stroke risk, and this effect becomes more pronounced with age. Furthermore, several diabetes-related interactions (such as age–diabetes, heart disease–diabetes, and hypertension–diabetes) also showed significant trends, reflecting the synergistic effect of metabolic diseases under multiple cardiovascular risk conditions.

### 3.5 Training resources and complexity

**Figure 7:**
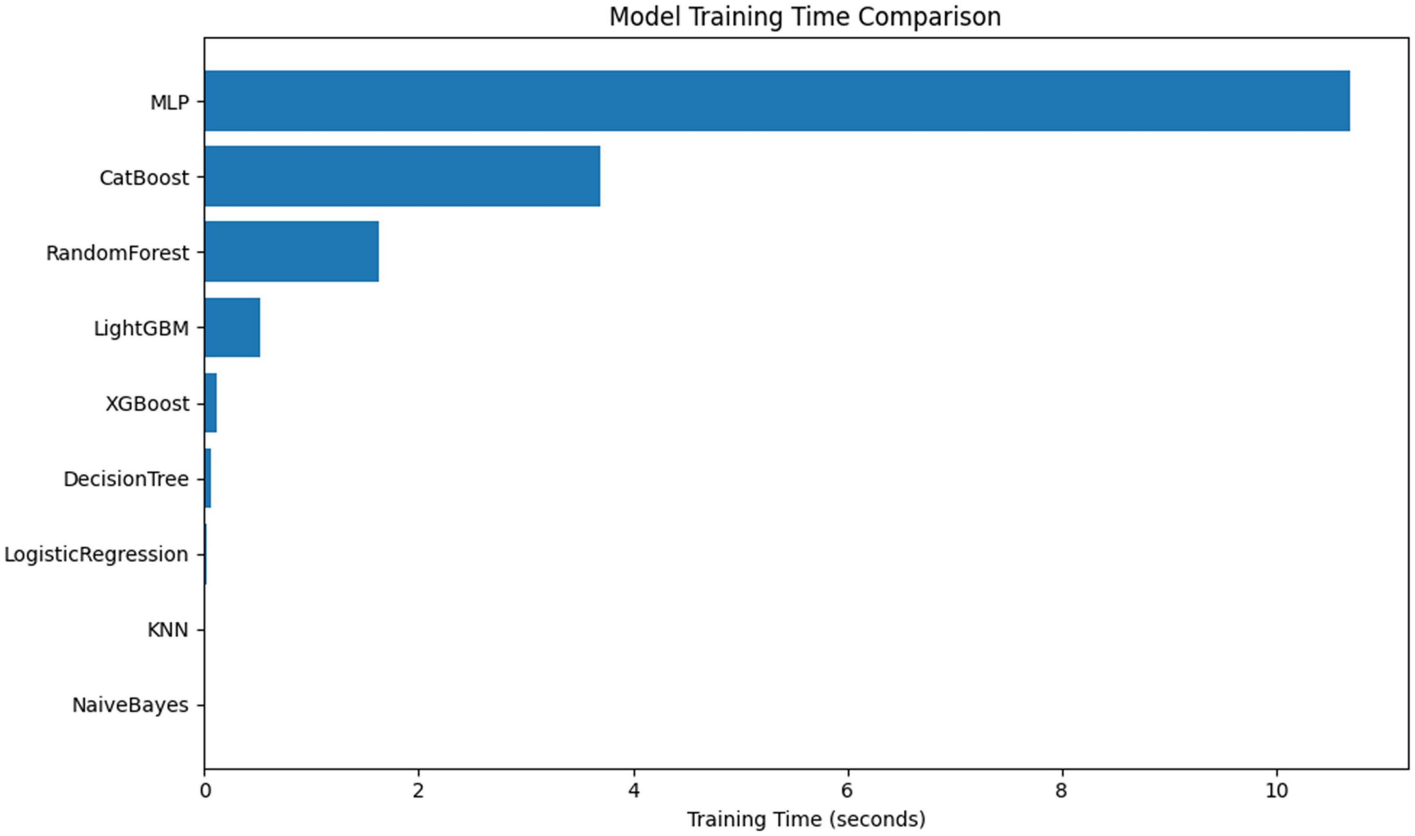
Comparison of model training time and parameter quantity.

Comparison of training time and parameter size of each model (Figure 7). MLP and AutoML have the longest training time; AutoML involves multiple rounds of search and fusion, and the deployment cost is high in resource-constrained scenarios. LR, NB, and KNN train quickly and are suitable for low-resource environments; RF and XGB maintain good performance under moderate resource conditions and are more balanced choices (35).

## 4 Discussion

Based on the above results, CatBoost and AutoML demonstrated the best overall performance. CatBoost has strong interpretability and resistance to overfitting, making it more suitable as a medical decision support tool; AutoML, on the other hand, can be quickly deployed with almost no professional background due to its high degree of automation. SHAP’s global and individual-level interpretations are largely consistent with clinical experience, revealing the intersection between the model’s focus and human expert judgment, enhancing the model’s clinical acceptability and transparency.

From a model evaluation perspective, ROC and PR are complementary in class imbalance scenarios. ROC can reflect the model’s overall discriminative ability, but it may suffer from the problem of “false positives”; in contrast, PR more accurately reflects the model’s ability to identify minority classes (stroke). In this study, Logistic Regression and LightGBM performed robustly in the PR space, followed by XGBoost and MLP; DT and KNN performed poorly. The confusion matrix further validated the differences between the various models and demonstrated the trade-offs between TP, FP, TN, and FN. This information directly corresponds to practical clinical needs, such as the costs of false positives or the risk of missed diagnoses. At the feature level, core variables such as age, hypertension, avg. glucose, BMI, and heart disease maintain consistent importance rankings across multiple models, consistent with previous evidence. Early screening of high-risk groups is crucial for stroke prevention. Age is the most important risk factor for stroke, and population aging makes this disease a major public health issue with a trend toward affecting younger people (38). As people age, they develop more vascular risk factors, and the incidence of stroke in men increases significantly, especially after age 35 (39). In addition, the longer an elderly person has had hypertension, the more types of medications they use, and the higher their systolic blood pressure, the greater the risk of stroke. Therefore, delaying the onset of hypertension can help reduce the risk of stroke (40). Elevated body mass index (BMI) leads to an increased incidence of stroke, especially among people over 40 years of age (41). Body mass index, blood glucose, and blood pressure are key metrics among “Life’s Essential 8” for assessing cardiovascular health, which is a significant predictor of stroke risk (42). A cohort study revealed that the risk of stroke is elevated across various types of coronary heart disease, and enhanced management of the disease course may effectively help prevent stroke (43). We also observed the sensitivity of MLP and XGBoost to social and environmental variables such as smoking status, SES, work type, and residence type, suggesting that in addition to physiological and metabolic indicators, social behavior and residential/occupational environment also affect stroke risk.

Beyond feature interpretation, our findings echo recent advancements in interpretable artificial intelligence for stroke and cardiovascular disease prediction. Existing research has demonstrated that combining machine learning models with interpretive methods such as SHAP can reliably identify clinically significant key risk factors, such as age, blood pressure, blood glucose, and BMI, playing a crucial role in improving model transparency and clinical trust. For example, a recently published cardiovascular disease prediction study utilized SHAP to construct an interpretability framework, successfully revealing risk patterns consistent with epidemiological evidence and supporting risk stratification in real-world scenarios (44). Our results are generally consistent with these works, while further advancing the depth of interpretation. In addition to demonstrating the global importance of features, we also identified multiple synergistic feature combinations through SHAP interaction analysis, such as hypertension–heart disease and BMI–avg glucose. These interactions are difficult to capture using univariate explanation methods, but they can reflect the risk changes under the combined effects of metabolic and cardiovascular risk factors, providing a more detailed and clinically consistent perspective for understanding the mechanisms of stroke.

Beyond calibration, the application of a model in real-world clinical scenarios also depends on the choice of decision threshold. In stroke risk prediction, false negatives often carry higher clinical risks. Therefore, in screening scenarios, a threshold biased toward higher recall (e.g., 0.30–0.40) can be appropriately used to minimize the omission of high-risk individuals. However, in situations with limited resources or high false positive costs, a more balanced threshold (e.g., 0.45–0.50) can be used to balance sensitivity and specificity. This result indicates that in practical applications, the risk probability output by the model needs to be interpreted and threshold settings should be tailored to specific clinical needs.

In terms of model deployment and resource consumption, deep models and AutoML offer performance advantages and high automation, but the training and inference processes require more computational resources. In contrast, LR, NB, and KNN are more lightweight and efficient; RF and XGBoost achieve a better balance between performance and resource requirements, making them suitable for resource-constrained applications that still need to maintain high accuracy. LR/NB/KNN are lightweight and efficient. RF/XGB offers a better balance between performance and resources, making them suitable for scenarios where resources are limited but accuracy is required. Regarding method improvement and clinical implementation, the results suggest that strategies such as probability calibration, class resampling, and interpretability enhancement (SHAP/LIME) can be further combined to enhance the model’s usability and reliability in personalized stroke risk prediction. Regarding strategy selection, thresholds and cost-sensitive learning can be adjusted based on clinical priorities (reducing missed diagnoses vs. reducing false alarms) to meet specific scenario requirements.

## 5 Conclusion

Based on a public health indicator dataset, this study constructed and evaluated the applicability of multiple machine learning and deep learning models for stroke risk prediction and systematically compared their performance, interpretability, and resource consumption. The main conclusions are as follows:

Ensemble models (e.g., Voting, XGBoost, and CatBoost) significantly outperformed traditional models in multiple metrics (AUC, F1, and Recall) and demonstrated enhanced identification of high-risk individuals.

AutoML can automatically search for optimal model combinations, further improving predictive performance and providing an efficient path for clinical model selection.

SHAP analysis revealed the key role of variables such as age, hypertension, avg. glucose, and BMI in prediction, enhancing model interpretability and providing clear guidance for clinical screening.

Individual-level SHAP interpretations can be used for clinical risk communication and personalized intervention strategy design, with potential practical value.

In terms of training resources and complexity, tree models and deep models each have advantages, and trade-offs can be made based on the scenario.

This study demonstrates a comprehensive “multi-model prediction + interpretable analysis” framework, enhancing the usability and transparency of machine learning in clinical decision-making and demonstrating that health indicator-based prediction models can be used for screening high-risk populations. The results are expected to provide theoretical support and technical reference for early stroke screening, community health interventions, and the development of digital health tools.

## Data Availability

The original contributions presented in the study are included in the article/Supplementary material, further inquiries can be directed to the corresponding author/s.

## Footnotes

1
https://www.kaggle.com/datasets/shriyashjagtap/stroke-diagnosis-and-health-metrics-data

## References

[1] Lo EH Dalkara T Moskowitz MA. Mechanisms, challenges and opportunities in stroke. Nat Rev Neurosci. (2003) 4:399–414. doi: 10.1038/nrn1106, 12728267

[2] Gorelick PB. The global burden of stroke: persistent and disabling. Lancet Neurol. (2019) 18:417–8. doi: 10.1016/S1474-4422(19)30030-4, 30871943

[3] Liu X Hao Y Huang Z Shi Y Su C Zhao L. Modulation of microglial polarization by sequential targeting surface-engineered exosomes improves therapy for ischemic stroke. Drug Deliv Transl Res. (2024) 14:418–32. doi: 10.1007/s13346-023-01408-6, 37587291

[4] Meschia JF Bushnell C Boden-Albala B Braun LT Bravata DM Chaturvedi S . Guidelines for the primary prevention of stroke. Stroke. (2014) 45:3754–832. doi: 10.1161/STR.0000000000000046, 25355838 PMC5020564

[5] Lanas F Seron P. Facing the stroke burden worldwide. Lancet Glob Health. (2021) 9:e235–6. doi: 10.1016/S2214-109X(20)30520-9 33422188

[6] Rajkomar A Dean J Kohane I. Machine learning in medicine. N Engl J Med. (2019) 380:1347–58. doi: 10.1056/NEJMra1814259 30943338

[7] Beam AL Kohane IS. Big data and machine learning in health care. JAMA. (2018) 319:1317–8. doi: 10.1001/jama.2017.18391, 29532063

[8] Saraswat D Bhattacharya P Verma A Prasad VK Tanwar S Sharma G . Explainable AI for healthcare 5.0: opportunities and challenges. IEEE Access. (2022) 10:84486–517. doi: 10.1109/ACCESS.2022.3197671

[9] Breiman L. Random forests. Mach Learn. (2001) 45:5–32. doi: 10.1023/A:1010933404324, 40797221

[10] Chen T Guestrin C. *XGBoost: a scalable tree boosting system*. In: Proceedings of the 22nd ACM SIGKDD International Conference on Knowledge Discovery and Data Mining, 2016. San Francisco, CA: Association for Computing Machinery, pp. 785–794. (2016).

[11] Kumar PS Kumari A Mohapatra S Naik B Nayak J Mishra M. *CatBoost ensemble approach for diabetes risk prediction at early stages*. 2021 1st Odisha international conference on electrical power engineering, communication and computing technology (ODICON). IEEE, pp. 1–6 (2021).

[12] Cutler DR Edwards TC Jr Beard KH Cutler A Hess KT Gibson J . Random forests for classification in ECOLOGY. Ecology. (2007) 88:2783–92. doi: 10.1890/07-0539.1, 18051647

[13] Fahrenthold BK Cavanaugh MR Jang S Murphy AJ Ajina S Bridge H . Optic tract shrinkage limits visual restoration after occipital stroke. Stroke. (2021) 52:3642–50. doi: 10.1161/STROKEAHA.121.034738, 34266305 PMC8545836

[14] Svetnik V Liaw A Tong C Culberson JC Sheridan RP Feuston BP. Random Forest: a classification and regression tool for compound classification and QSAR modeling. J Chem Inf Comput Sci. (2003) 43:1947–58. doi: 10.1021/ci034160g, 14632445

[15] Friedman JH. Greedy function approximation: a gradient boosting machine. Ann Stat. (2001) 29:1189–232. doi: 10.1214/aos/1013203451

[16] Figueroa A. Automatically generating effective search queries directly from community question-answering questions for finding related questions. Expert Syst Appl. (2017) 77:11–9. doi: 10.1016/j.eswa.2017.01.041

[17] Schmidhuber J. Deep learning in neural networks: an overview. Neural Netw. (2015) 61:85–117. doi: 10.1016/j.neunet.2014.09.003 25462637

[18] LeCun Y Bengio Y Hinton G. Deep learning. Nature. (2015) 521:436–44. doi: 10.1038/nature14539, 26017442

[19] Elgendy M Sik-Lanyi C Kelemen A. A novel marker detection system for people with visual impairment using the improved tiny-YOLOv3 model. Comput Methods Prog Biomed. (2021) 205:106112. doi: 10.1016/j.cmpb.2021.106112, 33915507

[20] Dritsas E Trigka M. Stroke risk prediction with machine learning techniques. Sensors. (2022) 22:670. doi: 10.3390/s22134670, 35808172 PMC9268898

[21] Heo J Yoon JG Park H Kim YD Nam HS Heo JH. Machine learning–based model for prediction of outcomes in acute stroke. Stroke. (2019) 50:1263–5. doi: 10.1161/STROKEAHA.118.024293, 30890116

[22] Noh Y Lee H Choi A Kwon JS Choe S-A Chae J . First-trimester exposure to benzodiazepines and risk of congenital malformations in offspring: a population-based cohort study in South Korea. PLoS Med. (2022) 19:e1003945. doi: 10.1371/journal.pmed.1003945, 35235572 PMC8926183

[23] Ethan Tan K Sesagiri Raamkumar A Wee HL. Impact of COVID-19 on the outreach strategy of cancer social service agencies in Singapore: a pre-post analysis with Facebook data. J Biomed Inform. (2021) 118:103798. doi: 10.1016/j.jbi.2021.103798, 33965641 PMC9155955

[24] Son DY Kwon HB Lee DS Jin HW Jeong JH Kim J . Changes in physiological network connectivity of body system in narcolepsy during REM sleep. Comput Biol Med. (2021) 136:104762. doi: 10.1016/j.compbiomed.2021.104762 34399195

[25] Marker DA Mardon R Jenkins F Campione J Nooney J Li J . State-level estimation of diabetes and prediabetes prevalence: combining national and local survey data and clinical data. Stat Med. (2018) 37:3975–90. doi: 10.1002/sim.7848, 29931829

[26] Li J Luo Y Dong M Liang Y Zhao X Zhang Y . Tree-based risk factor identification and stroke level prediction in stroke cohort study. Biomed Res Int. (2023) 2023:191. doi: 10.1155/2023/7352191, 37078009 PMC10110369

[27] Saito T Rehmsmeier M. The precision-recall plot is more informative than the ROC plot when evaluating binary classifiers on imbalanced datasets. PLoS One. (2015) 10:e0118432. doi: 10.1371/journal.pone.0118432, 25738806 PMC4349800

[28] Ribeiro MT Singh S Guestrin C. *“Why should I trust you?”: Explaining the predictions of any classifier*. In: Proceedings of the 22nd ACM SIGKDD International Conference on Knowledge Discovery and Data Mining, San Francisco, CA: Association for Computing Machinery, pp. 1135–1144. (2016).

[29] Genocchi B Lenk K Hyttinen J. Influence of astrocytic gap junction coupling on in silico neuronal network activity In: Genocchi B, editor. Mediterranean Conference on Medical and Biological Engineering and Computing. Berlin: Springer (2019). 480–7.

[30] Nakajima K Tokita Y Tanaka A Takahashi S. The VLDL receptor plays a key role in the metabolism of postprandial remnant lipoproteins. Clin Chim Acta. (2019) 495:382–93. doi: 10.1016/j.cca.2019.05.004, 31078566

[31] Liu Y Fu Y Peng Y Ming J. Clinical decision support tool for breast cancer recurrence prediction using SHAP value in cooperative game theory. Heliyon. (2024) 10:e24876. doi: 10.1016/j.heliyon.2024.e24876, 38312672 PMC10835316

[32] Chen S Ren C Zhai J Yu J Zhao X Li Z . CAFU: a galaxy framework for exploring unmapped RNA-Seq data. Brief Bioinform. (2020) 21:676–86. doi: 10.1093/bib/bbz018, 30815667 PMC7299299

[33] Gurven M Blackwell AD Rodríguez DE Stieglitz J Kaplan H. Does blood pressure inevitably rise with age? Hypertension. (2012) 60:25–33. doi: 10.1161/HYPERTENSIONAHA.111.189100, 22700319 PMC3392307

[34] Fletcher R Wilkinson E Cleary P Blagden S Farmer S. Did school characteristics affect the uptake of meningococcal quadrivalent vaccine in greater Manchester, United Kingdom? Public Health. (2019) 171:24–30. doi: 10.1016/j.puhe.2019.03.018, 31082757

[35] Vu T Kokubo Y Inoue M Yamamoto M Mohsen A Martin-Morales A . Machine learning approaches for stroke risk prediction: findings from the Suita study. J Cardiovasc Dev Dis. (2024) 11:207. doi: 10.3390/jcdd11070207, 39057627 PMC11276746

[36] Klug J Leclerc G Dirren E Carrera E. Machine learning for early dynamic prediction of functional outcome after stroke. Commun Med. (2024) 4:232. doi: 10.1038/s43856-024-00666-w, 39537988 PMC11561255

[37] Zhuo X Lv J Chen B Liu J Luo Y Liu J . Combining conventional ultrasound and ultrasound elastography to predict HER2 status in patients with breast cancer. Front Physiol. (2023) 14:502. doi: 10.3389/fphys.2023.1188502 PMC10369848 37501928

[38] BroderickJP. Revolution in stroke treatment over 50 years and predicting stroke care in 2050. Stroke. (2025) 2025:2583. doi: 10.1161/STROKEAHA.125.05258341099129

[39] Sturzenegger R. First ischemic stroke in young adults: Sex and age-related differences in stroke rates, risk factors, and etiologies. Eur Stroke J. (2025) 10:882–91. doi: 10.1177/23969873251317347, 39916317 PMC11803591

[40] Howard G Muntner P Lackland DT Plante TB Cushman M Stamm B . Association of Duration of recognized hypertension and stroke risk: the REGARDS study. Stroke. (2025) 56:105–12. doi: 10.1161/STROKEAHA.124.048385, 39648907 PMC11661922

[41] ShiY QianC LinX ZhuY. Association between a body shape index and the prevalence of stroke: a cross-sectional study. Neuroepidemiology. (2025) 2025:1–14. doi: 10.1159/000549080, 41144613

[42] Wu S Wu Z Yu D Chen S Wang A Wang A . Life’s essential 8 and risk of stroke: a prospective community-based study. Stroke. (2023) 54:2369–79. doi: 10.1161/STROKEAHA.123.042525, 37466001

[43] Sodhi-Berry N Burchill LJ Kleinig TJ Nedkoff L Katzenellenbogen JM. Incidence and predictors of stroke in Australian adults with congenital heart disease (2000–2017). J Am Heart Assoc. (2024) 13:e034057. doi: 10.1161/JAHA.123.034057, 39190566 PMC11646527

[44] Lv J Zhang M Fu Y Chen M Chen B Xu Z . An interpretable machine learning approach for predicting 30-day readmission after stroke. Int J Med Inform. (2023) 174:105050. doi: 10.1016/j.ijmedinf.2023.105050, 36965404

