# Supplementary material for: Explainable machine learning for stroke risk prediction: a comparative study with SHAP-based interpretation
Source: Front Neurol. 2026 Jan 12;16:1716984. doi: 10.3389/fneur.2025.1716984 (PMC12832496; doi:10.3389/fneur.2025.1716984)
Supplement: Supplementary file 1 [file Table_1.DOCX]

| Feature | Type | Non-missing samples | | Missing values | unique values | common value | Mean | Standard Deviation |
| --- | --- | --- | --- | --- | --- | --- | --- | --- |
| Age | Continuous | 10000 | 0 | | 81 | 57.0 | 43.2 | 22.2 |
| BMI | Continuous | 9700 | 300 | | 418 | 28.6 | 28.9 | 7.5 |
| Avg Glucose Level | Continuous | 10000 | 0 | | 3566 | 93.88 | 106.1 | 46.5 |
| Gender | Category | 10000 | 0 | | 2 | Female |  |  |
| Hypertension | Category | 10000 | 0 | | 2 | 0 |  |  |
| Heart Disease | Category | 10000 | 0 | | 2 | 0 |  |  |
| Ever Married | Category | 10000 | 0 | | 2 | Yes |  |  |
| Work Type | Category | 10000 | 0 | | 5 | Private |  |  |
| Residence Type | Category | 10000 | 0 | | 2 | Urban |  |  |
| Smoking Status | Category | 9500 | 500 | | 4 | never smoked | |  |
| SES | Category | 10000 | 0 | | 5 | Upper |  |  |

**Supplementary Table. 1.** Descriptive statistics of all variables included in the study.

| **Model** | **Hyperparameter Search Range** | **Final Selected Parameters** |
| --- | --- | --- |
| Logistic Regression (LR) | C ∈ {0.01, 0.1, 1, 10}; penalty ∈ {L2} | C = 1.0; penalty = L2 |
| K-Nearest Neighbor (KNN) | N neighbors ∈ {3, 5, 7, 11}; weights ∈ {uniform, distance} | N neighbors = 7; weights = distance |
| Decision Tree (DT) | Max depth ∈ {3, 5, 10, None}; min samples split ∈ {2, 5, 10} | Max depth = 10; min samples split = 2 |
| Naive Bayes (NB) | - | default |
| Support Vector Machine (SVM, RBF) | C ∈ {0.1, 1, 10}; gamma ∈ {0.01, 0.1, 1}; kernel ∈ {rbf, linear} | C = 1; gamma = 0.1; kernel = rbf |
| Random Forest (RF) | N estimators ∈ {100, 200, 300}; max depth ∈ {None, 10, 20}; min samples split ∈ {2, 5} | N estimators = 300; max depth = None; min samples split = 2 |
| XGBoost (XGB) | N estimators ∈ {200, 400, 600}; max depth ∈ {3, 5, 7}; learning rate ∈ {0.01, 0.05, 0.1}; subsample ∈ {0.7, 1.0} | N estimators = 400; max depth = 5; learning rate = 0.05; subsample = 0.7 |
| LightGBM (LGBM) | N estimators ∈ {200–800}; num leaves ∈ {15–50}; learning rate ∈ {0.01–0.1}; max depth ∈ {−1, 5, 10} | N estimators = 500; num leaves = 31; learning rate = 0.03 |
| CatBoost (CB) | depth ∈ {4–10}; iterations ∈ {300–800}; learning rate ∈ {0.01–0.1}; l2 leaf reg ∈ {1, 3, 5} | depth = 6; iterations = 500; learning rate = 0.03; l2 leaf reg = 3 |
| Multi-Layer Perceptron (MLP) | Hidden layer sizes ∈ {(64,), (128,), (64,32)}; activation ∈ {relu, tanh}; alpha ∈ {0.0001–0.01} | Hidden layer sizes = (64,32); activation = relu; alpha = 0.0001 |
| Voting Classifier | base estimators ∈ {LR, RF, XGB…}; voting ∈ {soft, hard} | soft voting; estimators = LR+RF+XGB |
| Stacking Classifier | base-models ∈ {LR, RF, XGB}; meta-model ∈ {LR, RF} | base-models = LR+RF+XGB; meta-model = LR |
| AutoML Framework | - | - |

**Supplementary Table 2.** Hyperparameter search ranges and final selected parameters for all benchmarked machine learning models.

|  | Age | Hypertension | Heart Disease | BMI | Avg Glucose | Diabetes | Stroke | Gender Female | Gender Male | SES High | SES Low | SES Medium | Smoking Status Current | Smoking Status Former | Smoking Status Never |
| --- | --- | --- | --- | --- | --- | --- | --- | --- | --- | --- | --- | --- | --- | --- | --- |
| Age | 1 | 0.417468 | 0.109534 | -0.00132 | 0.138313 | 0.132659 | 0.362514 | -0.00708 | 0.00708 | 0.006393 | -0.02748 | 0.019734 | 0.004871 | 0.001136 | -0.00494 |
| Hypertension | 0.417468 | 1 | 0.051501 | -0.00369 | 0.308235 | 0.058061 | 0.388837 | -0.01163 | 0.011629 | 0.009606 | -0.02382 | 0.01383 | 0.009529 | -0.00088 | -0.00714 |
| Heart Disease | 0.109534 | 0.051501 | 1 | -0.00234 | 0.297635 | 0.022211 | 0.274265 | 0.008182 | -0.00818 | 0.011989 | -0.02552 | 0.013455 | 0.000337 | -0.00909 | 0.007111 |
| BMI | -0.00132 | -0.00369 | -0.00234 | 1 | 0.005738 | -0.00392 | 0.083252 | 0.003549 | -0.00355 | -0.00615 | 0.01017 | -0.00426 | -0.00791 | 0.003371 | 0.003779 |
| Avg Glucose | 0.138313 | 0.308235 | 0.297635 | 0.005738 | 1 | 0.009723 | 0.220624 | -0.0069 | 0.006903 | -0.00774 | -0.02215 | 0.026262 | 0.006719 | 0.000102 | -0.00562 |
| Diabetes | 0.132659 | 0.058061 | 0.022211 | -0.00392 | 0.009723 | 1 | 0.248065 | 0.008942 | -0.00894 | 0.014606 | -0.00419 | -0.00795 | 0.012358 | 0.001609 | -0.01149 |
| Stroke | 0.362514 | 0.388837 | 0.274265 | 0.083252 | 0.220624 | 0.248065 | 1 | -0.00482 | 0.004819 | 0.007213 | -0.02692 | 0.018563 | 0.079962 | -0.03493 | -0.03751 |
| Gender Female | -0.00708 | -0.01163 | 0.008182 | 0.003549 | -0.0069 | 0.008942 | -0.00482 | 1 | -1 | 0.002743 | 0.005916 | -0.00756 | -0.02256 | 7.42E-05 | 0.018531 |
| Gender Male | 0.00708 | 0.011629 | -0.00818 | -0.00355 | 0.006903 | -0.00894 | 0.004819 | -1 | 1 | -0.00274 | -0.00592 | 0.007559 | 0.022557 | -7.4E-05 | -0.01853 |
| SES High | 0.006393 | 0.009606 | 0.011989 | -0.00615 | -0.00774 | 0.014606 | 0.007213 | 0.002743 | -0.00274 | 1 | -0.3196 | -0.51454 | 0.004707 | -0.00884 | 0.003305 |
| SES Low | -0.02748 | -0.02382 | -0.02552 | 0.01017 | -0.02215 | -0.00419 | -0.02692 | 0.005916 | -0.00592 | -0.3196 | 1 | -0.64804 | -0.00972 | -0.0007 | 0.008585 |
| SES Medium | 0.019734 | 0.01383 | 0.013455 | -0.00426 | 0.026262 | -0.00795 | 0.018563 | -0.00756 | 0.007559 | -0.51454 | -0.64804 | 1 | 0.005015 | 0.00774 | -0.01042 |
| Smoking Status Current | 0.004871 | 0.009529 | 0.000337 | -0.00791 | 0.006719 | 0.012358 | 0.079962 | -0.02256 | 0.022557 | 0.004707 | -0.00972 | 0.005015 | 1 | -0.2538 | -0.61789 |
| Smoking Status Former | 0.001136 | -0.00088 | -0.00909 | 0.003371 | 0.000102 | 0.001609 | -0.03493 | 7.42E-05 | -7.4E-05 | -0.00884 | -0.0007 | 0.00774 | -0.2538 | 1 | -0.6037 |
| Smoking Status Never | -0.00494 | -0.00714 | 0.007111 | 0.003779 | -0.00562 | -0.01149 | -0.03751 | 0.018531 | -0.01853 | 0.003305 | 0.008585 | -0.01042 | -0.61789 | -0.6037 | 1 |

**Supplementary Table. 3.** Spearman correlation matrix among all study variables.

| **Model** | **Accuracy** | **F1** | **Recall** | **AUC** | **PR-AUC** |
| --- | --- | --- | --- | --- | --- |
| LR | 0.84 | 0.42 | 0.38 | 0.85 | 0.37 |
| KNN | 0.82 | 0.39 | 0.35 | 0.83 | 0.34 |
| DT | 0.79 | 0.35 | 0.3 | 0.8 | 0.3 |
| RF | 0.87 | 0.5 | 0.45 | 0.89 | 0.48 |
| XGBoost | 0.88 | 0.52 | 0.48 | 0.91 | 0.5 |
| LightGBM | 0.87 | 0.51 | 0.47 | 0.9 | 0.49 |
| CatBoost | 0.88 | 0.54 | 0.49 | 0.92 | 0.52 |
| MLP | 0.86 | 0.48 | 0.44 | 0.89 | 0.47 |
| Voting (soft) | 0.89 | 0.56 | 0.51 | 0.93 | 0.54 |
| AutoML | 0.89 | 0.57 | 0.52 | 0.94 | 0.55 |

**Supplementary Table. 4.** Performance of all machine learning models on the independent test set.

| **Model** | **AUC mean** | **AUC sd** | **PR AUC mean** | **PR AUC sd** | **F1 mean** | **F1 sd** | **Recall mean** | **Recall sd** |
| --- | --- | --- | --- | --- | --- | --- | --- | --- |
| LR | 0.849306 | 0.011589 | 0.727823 | 0.009935 | 0.660618 | 0.014184 | 0.766572 | 0.013414 |
| KNN | 0.712196 | 0.021992 | 0.479325 | 0.024124 | 0.532359 | 0.022304 | 0.618811 | 0.029312 |
| DT | 0.659279 | 0.015199 | 0.413995 | 0.016378 | 0.522336 | 0.020865 | 0.527706 | 0.019931 |
| RF | 0.822271 | 0.012088 | 0.6928 | 0.009859 | 0.595163 | 0.012845 | 0.541546 | 0.018689 |
| XGBoost | 0.845026 | 0.011494 | 0.732865 | 0.011936 | 0.641333 | 0.017101 | 0.598649 | 0.027184 |
| LightGBM | 0.835365 | 0.009233 | 0.717177 | 0.010634 | 0.614018 | 0.01023 | 0.557936 | 0.014304 |
| CatBoost | 0.849044 | 0.010743 | 0.736478 | 0.012589 | 0.640323 | 0.020188 | 0.594446 | 0.032029 |
| MLP | 0.844826 | 0.010334 | 0.719212 | 0.011736 | 0.651056 | 0.022891 | 0.772919 | 0.049949 |
| Voting (soft) | 0.846836 | 0.011824 | 0.731808 | 0.009354 | 0.653819 | 0.017305 | 0.649017 | 0.025092 |
| AutoML | 0.844904 | 0.012135 | 0.73029 | 0.010504 | 0.649533 | 0.01663 | 0.637682 | 0.023922 |

**Supplementary Table 5.** Five-fold stratified cross-validation results of all machine learning models (mean ± SD).

| Feature 1 | Feature 2 | Interaction Strength |
| --- | --- | --- |
| Gender Female | Gender Male | 0.092241 |
| Hypertension | Heart Disease | 0.085964 |
| Age | Heart Disease | 0.069083 |
| Age | Hypertension | 0.062236 |
| Age | Diabetes | 0.039708 |
| Age | BMI | 0.030004 |
| BMI | Avg Glucose | 0.02985 |
| Age | Avg Glucose | 0.024784 |
| Heart Disease | Diabetes | 0.024474 |
| Hypertension | Diabetes | 0.020739 |

**Supplementary Table 6.** Top 10 strongest SHAP interaction effects identified by the XGBoost model.
